# Role of long non-coding RNA H19 in therapy resistance of digestive system cancers

**DOI:** 10.1186/s10020-020-00255-2

**Published:** 2021-01-05

**Authors:** Jingting Wang, Xiao Ma, Hai Si, Zhongjun Ma, Yan Ma, Jing Wang, Bangwei Cao

**Affiliations:** 1 Department of Oncology, Beijing Friendship Hospital, Capital Medical University, #95 Yong An Road, Xicheng District, Beijing, 100050 China; 2 Department of Comprehensive Medicine, Beijing Shijingshan Hospital, #24 Shijingshan Road, Shijingshan District, Beijing, 100043 China; 3 Yale School of Medicine, New Haven, CT USA

**Keywords:** LncRNA-H19, Digestive system cancer, Chemoresistant, Radioresistant

## Abstract

Digestive system cancers are associated with high morbidity and mortality. Chemotherapy and radiotherapy are the main treatment modalities for these cancers. However, the development of therapy resistance leads to high rates of tumor recurrence and metastasis, resulting in dismal prognosis. Long non-coding RNA (LncRNA) H19, one of the most intriguing non-coding RNAs, has been shown to play a key role in the development and therapy resistance of various digestive system cancers (including hepatocellular carcinoma, colorectal cancer, pancreatic ductal adenocarcinoma, esophageal carcinoma, gastric cancer, and biliary system cancer) by regulating the abnormal expression of genes. In this review, we discuss the potential mechanisms of LncRNA H19 related therapy resistance in the context of digestive system cancers. LncRNA H19 is a potential novel therapeutic target for amelioration of cancer therapy resistance.

## Introduction

Recent advances in the exploration of the human genome have shown that only 2% of all human genes are protein-coding genes, while the remaining genes are transcribed into non-coding RNAs. Depending on their length, non-coding RNAs are classified as long non-coding RNA (LncRNA), small nuclear RNA, small nucleolar RNA, micro RNA (miRNA), piwi interacting RNA, and small interfering RNA. The non-coding RNAs exhibit remarkable biological functions that involve a wide range of metabolic processes (Cech and Steitz 2014). LncRNAs with transcript length of > 200 nucleotides play a key role in multiple pathophysiological processes involved in carcinogenesis, including cell proliferation, differentiation, metastasis, angiogenesis, and therapy resistance (Ghafouri-Fard et al. 2020; Guzel et al. 2019; Lin et al. 2020; Pan et al. 2020; Teppan et al. 2020; Zhang et al. 2020). LncH19 was the first discovered LncRNA; it is located on human chromosome 11p15.5 and has a total length of 2.3 kb (contains 5 exons and 4 introns) (Cai and Cullen 2007; Ghafouri-Fard et al. 2020). As the imprinting gene, H19 mainly expresses maternal genes and is closely linked to the insulin growth factor 2 gene of the parental imprinting gene (Thorvaldsen et al. 1998). Generally, H19 exists in the cytoplasm and functions by regulating RNA or ribosomes (Schoenfelder et al. 2007). Several recent studies have identified abnormal expression of H19 in various human cancers, such as colorectal (Wu et al. 2017), liver (Tsang and Kwok 2007), gastric (Ishii et al. 2017), pancreatic (Yoshimura et al. 2018), esophageal (Li et al. 2019), breast (Zhu et al. 2017), lung (Li et al. 2019), glioma (Jia et al. 2018), ovarian (Wu et al. 2019), and hematological cancers (Yang et al. 2020). In addition, its gene polymorphism is closely related to cancer susceptibility (Li et al. 2020). Recent years have witnessed rapid advances in the treatment of digestive system cancers; however, the phenomenon of multidrug resistance (MDR) caused by long-term anti-cancer therapy has a detrimental effect on the treatment outcomes and prognosis of patients. Several mechanisms have been implicated in the development of MDR; these include, activation of energy-dependent transmembrane transporter with drug pump function, inhibition of apoptosis pathway, enhancement of DNA repair, regulation of tumor microenvironment, induction of autophagy, and detoxification of intracellular drugs (Jiang et al. 2020).

A plethora of recent studies have demonstrated the relationship between H19 and their specific modulated targets/pathways in digestive system cancers, such as induction of epithelial–mesenchymal transition (EMT), interference with apoptosis, regulation of the expression of MDR genes, and transfer of exosomes (Ren et al. 2018; Li et al. 2018). The major functions and functional mechanisms of H19 are summarized in Table 1. In this article, we focus exclusively on the molecular mechanism of therapeutic resistance in the context of digestive system cancers and highlight the potential contribution of H19 to the development of resistance to chemotherapy and radiotherapy.

**Table 1:** lncRNA-H19 and therapy resistance of digestive system cancers

Cancers	Cell samples	Expression in resistant cell	Biological mechanisms	Targets	Drugs	References
HCC	R-HepG2	High	Regulating methylation of MDR1 promoter to induce P-gp expression; Knock-down of H19 inhibited the expression of MDR1/P-gp	H19-MDR1-P-gp	DOX	Tsang and Kwok (2007)
	HepG2-GR	High	Up-regulating the expression of CD90, CD44 and CD133	H19-CD90-CD44-CD133	GEM	Yang and Yu (2019)
	Bel-7402, HepG2, Hep3b, QGY-7703, SMMC-7721	No report	Targeting PSEN1 through the H19/mir-193a-3p axis	H19/miR-193a-3p/PSEN1	Chemotherapy (DOX, paclitaxel, vinorelbine, 5-FU) and radiotherapy (single-dose X-ray)	Ma et al. (2018)
	HepG2/ADM	High	Mediating the EMT process through P-gp, ZEB1 and EC	H19-P-gp-ZEB1-EC-EMT	DOX and DDP	Li (2019)
	HepG2, Plc/Prf5, Huh7	Low	Enhancing the cytotoxic effect of DOX or inhibiting cell proliferation		DOX and sorafenib	Schultheiss et al. (2017)
	CD133 + HuH7, 42 patients tissues	High	Knock-down of H19 blocked the MAPK/ERK signaling pathway, decreasing the expression of MDR1 and GST-Π	H19-MAPK/ERK-MDR1-GST-Π		Ding et al. (2018)
	18 patients tissues, Huh7, Hep3B, SNU-449, SNU-387	High	Knockdown of H19 sensitized HCC cells to sorafenib by downregulating miR-675 to suppress EMT	H19- miR-675- EMT	Sorafenib	Xu et al. (2020)
CRC	HCT8, 110 patients tissues	High	Mediating the SIRT1 dependent autophagy pathway by combining with miR-194-5p	H19-miR-194–5p-SIRT1	5-FU	Wang et al. (2018a, b)
	HCT116, SW480	High	Exosomes derived from CAFs transferred H19 to colorectal cancer cells; LncH19 competed for the adsorption of miR-141 and activated the Wnt/β-catenin pathway	H19-miR-141-Wnt/β-catenin	Oxaliplatin	Ren et al. (2018)
	HT-29-R	High	Activation of the Wnt/β-catenin pathway	Wnt/β-catenin	Methotrexate	Wu et al. (2017)
	LoVo	High	Upregulating the MDR1, MRP1 and BCRP resistant proteins	H19-MDR1-MRP1-BCRP	5-FU	Wang et al. (2018a, b)
PDAC	PANC-1	No report	No significant difference in the survival rate of cancer cells and expression of ABCG2, MRP1 and ABCC2 between the H19 overexpression group and low expression group		GEM, albumin paclitaxel and 5-FU	Sasaki et al. (2018)
	PANC-1	High	Promoting metastasis of pancreatic cancer		5-FU and abraxane	Yoshimura et al. (2018)
ESCA	KYSE150	High	Inhibition of H19 up-regulated mir-22-3p expression and down-regulated WNT1 to inhibit the proliferation and migration of cancer cells	H19/miR-22-3p/WNT1	Radiotherapy	Luo et al. (2019)
GC	39 patients tissues, MKN7	High	Inhibition of H19 reduced the survival rate of tumor cells and improved the sensitivity	H19/IGF2BP3	DOX	Ishii et al. (2017)
	SGC-7901/DDP	High	Decreasing the expression of FADD	H19/miR-675/FADD	DDP	Yan et al. (2017)
CCA	QBC939	High	Decreasing the cancer cell survival rate		GEM	Qiu (2017)

*lncRNAs* long non-coding RNAs, *miRNAs* microRNAs, *HCC* hepatocellular carcinoma, *MDR1* multidrug resistance-associated protein 1, *P-gp* P-glycoprotein, *DOX* doxorubicin, *GEM* gemcitabine, *PSEN1* presenilin 1, *5-FU* 5-fluorouracil, *EMT* epithelial–mesenchymal transition, *ADM* adriamycin, *ZEB1* zinc finger E-box binding homeobox 1, *EC* E-cadherin*, DDP* Cisplatin, *GST-II* glutathione *S*-transferase-II, *CRC* colorectal cancer, *SIRT1* silent information regulator 1, *CAFs* carcinoma-associated fibroblasts, *MRP1* Multidrug resistance-associated protein 1, *BCRP* Breast cancer resistance protein, *PDAC* pancreatic ductal adenocarcinoma, *ABCG2* ATP-binding cassette superfamily G number 2, *ABCC2* ATP-binding cassette subfamily C member 2, *ESCA* esophageal carcinoma, *GC* gastric cancer, *FADD* Fas-associated death domain, *CCA* cholangiocarcinoma

### Hepatocellular carcinoma (HCC)

**Fig. 1:**
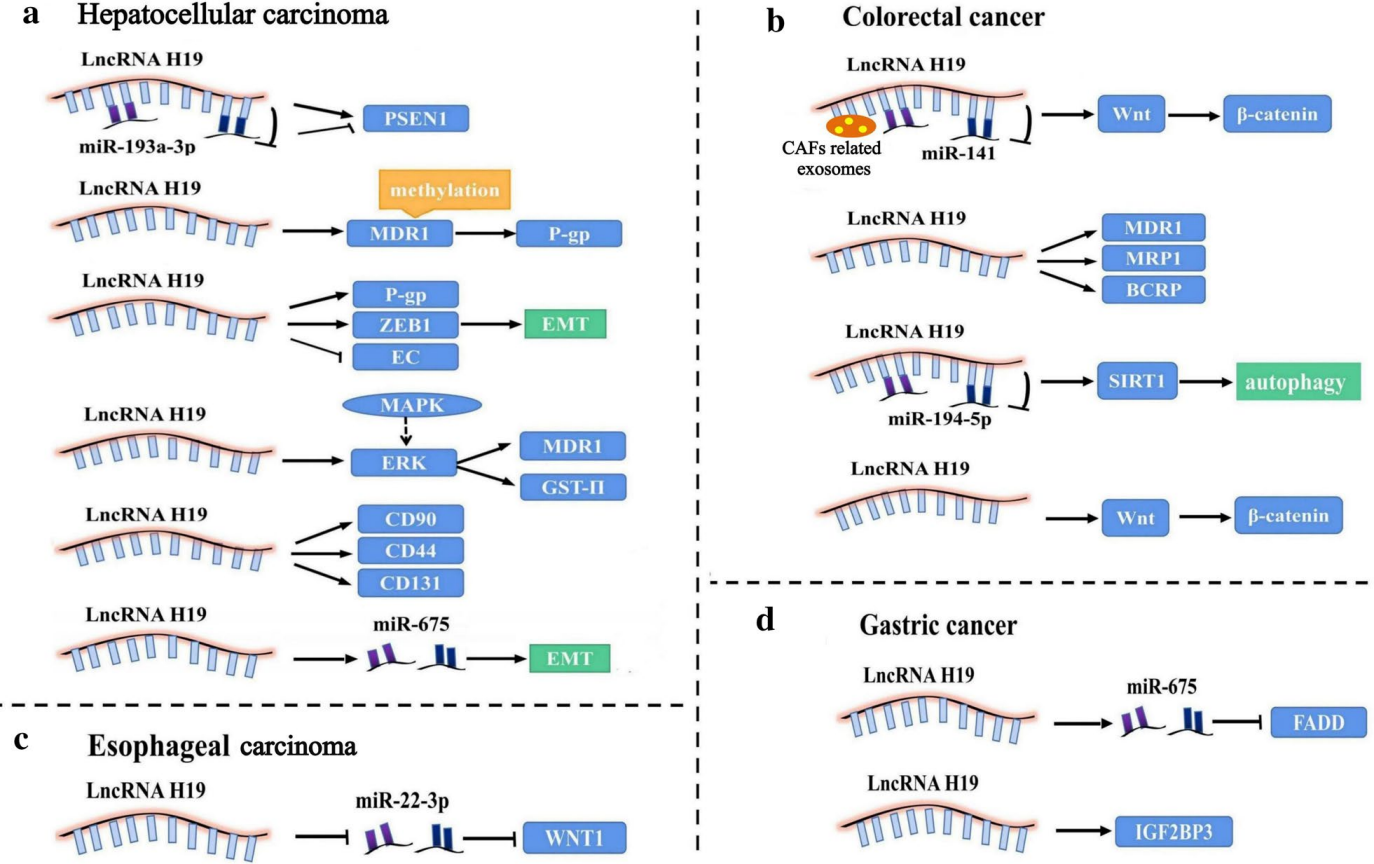
Overview of the role of H19 in modulating digestive system cancers therapy resistance. **a** LncH19 related pathways in hepatocellular carcinoma therapy resistance; **b** LncH19 related pathways in colorectal cancer chemoresistance; **c** LncH19 related pathways in esophageal carcinoma radioresistance; **d** LncH19 related pathways in gastric cancer chemoresistance

According to the Global Cancer Statistics 2018, liver cancer is now the sixth most frequently diagnosed cancer and the fourth leading cause of cancer deaths worldwide. HCC accounts for 75–85% of all cases of liver cancer (Bray et al. 2018). Currently, the main treatment modalities for primary HCC include chemotherapy, surgery, radiotherapy, targeted therapy, immunotherapy, and local ablative therapies. Patients with advanced unresectable HCC have been shown to benefit from combinations of chemotherapy and targeted therapies represented by sorafenib, lenvatinib, cisplatin, gemcitabine (GEM), 5-fluorouracil (5-FU), and doxorubicin (DOX) (Forner et al. 2018). However, patients with HCC are prone to develop resistance to conventional treatment, leading to relapse. A schematic illustration of the mechanisms by which H19 is involved in HCC therapy resistance is presented in Fig. 1a. Inhibition of H19 expression by antisense oligonucleotide transfection was shown to induce MDR1 promoter methylation and decrease the expression of multidrug resistance-associated protein 1 (MDR1) and its transcript P-glycoprotein (P-gp); this resulted in significant reduction in DOX 50% inhibition concentration (IC50) in R-HepG2 cells and enhanced their sensitivity to DOX (Tsang and Kwok 2007). H19 was also shown to be correlated with cisplatin resistance. Compared with the H19 low expression group, the IC50 of DOX and cisplatin was significantly greater in the H19 high expression group; in addition, the apoptosis rate in the low expression group (20.79 ± 2.22%) was significantly greater than that in the negative control group (4.16 ± 0.23%). According to the authors, antagonizing H19 diminished the expression of zinc finger E-box binding homeobox 1 (ZEB1) and P-gp, and upregulated the expression of E-cadherin (EC); thus, the chemotherapy resistance of HCC cells was reversed by blocking the EMT process (Li et al. 2019). In previous studies, GST-II was shown to promote chemotherapy resistance by influencing the biotransformation and metabolic processes (Liang 2010), and Ding’s research confirmed that down-regulation of H19 can block the MAPK/ERK signaling pathway, reducing the levels of MDR1 and GST-II; this was shown to facilitate cell apoptosis and suppress cell viability, eventually reversing the chemotherapy resistance of CD133 + HCC stem cells (Ding et al. 2018). Moreover, LncRNA H19 showed a close association with high expressions of HCC cancer stem cell markers (such as CD90, CD44, and CD133) and the generation of GEM resistance in HepG2 cell line. The IC50 of GEM was significantly lower after transfection with si-H19 (10.85 ± 2.19 vs 6.36 ± 1.54) (Yang and Yu 2019). Additionally, LncRNA H19 has been implicated in inducing radioresistance. For instance, in a study by Ma et al., lncRNA H19/miR-193a-3p axis was found to regulate the development and induction of radio-/chemo-resistance of HCC cells by targeting presenilin 1 (PSEN1). Restrained expression of lncRNA H19 and over-expression of miR-193a-3p tended to significantly increase the proliferation and survival rate of Bel-7402 cells, when these were tolerant to radiation (single-dose X-ray) and chemotherapeutic agents (DOX, paclitaxel, vinorelbine, 5-FU) (Ma et al. 2018). In addition to radiotherapy and chemotherapy, H19 has been shown to be involved in the resistance of HCC to the first targeted therapy, sorafenib. In the latest study on the relationship of H19 with sorafenib resistance, H19 expression in HCC tissue samples was significantly upregulated compared with normal tissues. Knockdown of H19 sensitized HCC cells to sorafenib by downregulating miR-675 and suppressing EMT (Xu et al. 2020). However, the role of H19 in therapy resistance of HCC is not completely elucidated. In contrast, H19 has also been shown to promote the sensitivity to chemotherapy. In the study by Schultheiss et al. (2017), H19 promoter methylation was significantly lesser in DOX resistant cells Plc/Prf/5 compared to their sensitive counterparts. By either increasing the cytotoxic action of DOX or by decreasing cell proliferation after sorafenib treatment, chemoresistant HCC cells were sensitized after H19 overexpression. Collectively, the above studies indicate the dual effect of H19 on therapy resistance in HCC. Further studies are required for better characterization of this dual effect.

### Colorectal cancer (CRC)

CRC is the third most common cancer in the world (Siegel et al. 2019) and the second most common cause of cancer-associated mortality (Bray et al. 2018). Currently, 5-FU based chemotherapy and novel target drugs such as cetuximab are recommended for patients with advanced CRC. Nevertheless, more than half of all patients develop metastasis and/or recurrence owing to chemotherapy resistance. The mechanism of chemotherapy resistance in CRC is mainly related to membrane transporters, abnormal DNA repair, apoptosis regulation, and signal transduction pathways (Ren et al. 2018; Wang et al. 2018a, b). The substantial role of H19 in carcinogenesis, progression, and chemotherapy resistance in the context of CRC has evoked considerable attention (Wu et al. 2017; Han et al. 2016), presented in Fig. 1b. According to a recent study (Ren et al. 2018), exosomes derived from carcinoma-associated fibroblasts (CAFs) transferred H19 to CRC cells, and H19 activated the downstream Wnt/β-catenin signaling pathway through competitive sponging of miR-141 to induce proliferation, invasion and metastasis of CRC stem cells; this promoted the stemness of CRC stem cells and induced oxaliplatin resistance in CRC cells in vitro and in vivo. In addition, activation of Wnt/β-catenin pathway by H19 overexpression was also shown to be involved in inducing resistance to methotrexate in HT-29-R cells (Wu et al. 2017). Interestingly, several studies have shown that H19 can interfere with the sensitivity of CRC cells to 5-FU in a variety of ways. For instance, IC50 of 5-FU in the overexpressed lncRNA H19 group was dramatically increased as compared to that in the H19 interfering group. The interfered H19 down-regulated the expressions of MDR1, multidrug resistance-associated protein 1 (MRP1) and breast cancer resistance protein (BCRP), which inhibited cell proliferation and migration, promoted apoptosis and reversed the sensitivity to 5-FU (Wang et al. 2018a, b). Different from the above mechanism, H19 can combine with miR-194-5p and mediate the silent information regulator 1 (SIRT1) dependent autophagy pathway to inhibit apoptosis of CRC cells and enhance their chemoresistance; this increased the IC50 of 5-Fu by 227.43% in H19 transfecting group compared with 5-FU sensitive cells HCT8 (Wang et al. 2018a, b). Thus, it is plausible that H19 induces acquired MDR in CRC patients, largely via its effect on mediating tumor apoptosis and migration.

### Pancreatic ductal adenocarcinoma (PDAC)

PDAC is a highly aggressive malignant tumor with an insidious onset and poor prognosis. The 5-year survival rate of PDAC patients is only 9%. It ranks as the seventh leading cause of cancer mortality in the world. The annual number of deaths is almost similar to the number of new cases (Bray et al. 2018; Siegel et al. 2019). Due to the rapid progression of PDAC and extremely low rate of satisfactory tumor resection, chemotherapy is the main treatment modality for PDAC. Thus, early containment of chemoresistance is worth pondering, especially in light of the discovery of the role of H19 in this field. In a study, PDAC cells treated with 5-FU or abraxane showed overexpression of H19 compared with non-treated cells, which suggested that H19 may be associated with drug resistance in pancreatic cancer cells (Yoshimura et al. 2018). In addition, H19 is not only a novel diagnostic and prognostic biomarker of PDAC, but also a promising therapeutic target (Wang et al. 2020). DTA-19 is a double-stranded DNA plasmid containing H19 regulatory sequence; it targets the highly expressed H19 in tumor cells to specifically kill cancer cells (Smaldone and Davies 2010). The tumoral volume in vitro after sequential administration of DTA-H19 and GEM was significantly lower than that observed after GEM monotherapy; this suggested that DTA-H19 enhances the antitumor activity of chemotherapy (Sorin et al. 2012). However, there is no clear consensus on the involvement of H19 in PDAC drug resistance. In a study by Sasaki et al. (2018), the survival rate of PDAC and expression of the members of ATP-binding cassette (ABC) transporters (such as ABC superfamily G number 2, MRP1 and ABC subfamily C member 2) showed no significant difference between the H19 overexpression group and H19 low expression group exposed to chemotherapy drugs (GEM, albumin paclitaxel, and 5-FU); this indicated that H19 may not be involved in mediating chemoresistance of PDAC. Currently, there is no direct evidence to confirm the relationship between H19 and pancreatic cancer drug resistance; therefore, further studies are required to explore the relationship between H19 and PDAC drug resistance.

### Esophageal carcinoma (ESCA)

Globally, an estimated 572,000 new cases of ESCA and approximately 509,000 deaths due to ESCA are reported each year (Bray et al. 2018). More than 80% of esophageal carcinomas are squamous cell carcinomas and are highly sensitive to radiotherapy. Radiation therapy is currently the standard treatment for unresectable ESCA (de Vos-Geelen et al. 2020). About 50% of patients develop local recurrence after concurrent radiotherapy and chemotherapy; increase in the radiation dose does not improve the therapeutic effect (Cooper et al. 1999; Minsky et al. 2002). Partial ESCA cells often relapse or progress in the form of small lesions after radiotherapy, indicating the presence of radiation resistance (Jing et al. 2009). The role of H19 in conferring radioresistance has received wide attention (Fig. 1c). In ESCA radioresistant cells KYSE150R, knockdown of H19 downregulated the WNT1 via upregulating miR-22-3p expression, which caused the inhibition of cell migration, proliferation, and stemness (Luo et al. 2019). However, there is a paucity of evidence pertaining to the chemotherapy resistance of ESCA related to the mechanisms involving H19. So far, in a study, knockdown of lncRNA H19 repressed cell proliferation, migration, and EMT via the STAT3-EZH2-β-catenin pathway (Chen et al. 2019). In addition, EMT has been shown to be involved in the development of resistance to various chemotherapeutic agents (Du and Shim 2016). However, there is still no direct evidence to implicate H19 in the development of chemotherapy resistance of ESCA.

### Gastric cancer (GC)

GC is the fifth most common malignancy and the third leading cause of cancer-related deaths in the world (Bray et al. 2018). Chemotherapeutic resistance is a formidable challenge in the treatment of GC (Fig. 1d). In a study, compared to SGC-7901 cells, cisplatin resistant SGC-7901/DDP cells showed high expressions of H19 and miR-675 and low expression of Fas-associated death domain (FADD), which suppressed caspase8 and caspase3 in the caspase pathway and apoptosis; the findings suggested that H19/miR-675 may induce drug resistance by regulating the apoptosis of GC cells (Yan et al. 2017). Knockdown of H19 was shown to reduce the viability of GC cells MKN7 treated by DOX and alleviate chemoresistance; the effects were mediated via modulation of the H19-IGF2BP3 axis (Ishii et al. 2017). All the above studies have confirmed that H19 can induce GC chemotherapy resistance; therefore, H19 is a potential therapeutic target for future drug development.

### Biliary system cancer

The 5-year survival rates of patients with gallbladder cancer and cholangiocarcinoma (CCA) are only 9–18% and 16.4%, respectively (Lv et al. 2019). Chemotherapeutic resistance is a major problem in the treatment of biliary system cancer. After treatment with GEM, the survival rate of CCA cells QBC939 and the weight of the tumor were significantly lower in H19 high expression group; the findings suggested that high expression of H19 can increase the sensitivity of CCA cells to GEM (Qiu 2017). In addition, H19 was shown to contribute to the invasive growth of CCA cells by affecting the EMT process, leading to poor prognosis and promoting drug resistance (Xu et al. 2017). Moreover, overexpression of H19 in gallbladder cancer cells was shown to promote EMT and enhance cancer invasiveness by up-regulating the Twist-related protein 1 (Twist1) (Wang et al. 2016). EMT is known to promote chemoresistance of various tumors (Smith and Bhowmick 2016; Shibue and Weinberg 2017). Collectively, these findings suggest that H19 may mediate drug resistance of malignant tumors of the biliary system.

## Conclusions

In summary, an increasing number of studies have investigated the role of H19 in conferring drug resistance of digestive system cancers and explored the underlying mechanisms. Based on the studies discussed in this review, H19 seems to induce drug resistance in ESCA, CRC, GC, and gallbladder cancer; however, it has a dual effect in promoting or inhibiting drug resistance in HCC, CCA, and PDAC. Based on the effect of H19 on drug resistance of individual cancers, H19 regulatory sequence with high or low expression may serve as a potential therapeutic target to reverse or hinder the occurrence of drug resistance. We speculate that the varied effects of H19 may be related to the differences in pathological type of tumors, the heterogeneity of the tumor cell lines, the cancer microenvironment, the drugs used, the downstream signaling pathway activated by H19, the specific experiment settings, and the nature of experiments (in vitro or in vivo). At present, LncRNA H19 in combination with chemotherapy has been shown to improve in vitro treatment efficacy against non-small-cell lung cancer (Zhou and Zhang 2020). The DTA-19 targeted by H19 has also shown promising prospects in anti-cancer therapy by suppressing tumor growth after intratumoral injection (Mizrahi et al. 2009). Nevertheless, there are certain challenges and inconsistencies in the available evidence. Due to the dual role of H19 in the context of different tumors, further research is required to provide more definitive evidence of the role of H19 and its determinants. Till date, most studies that have investigated the chemoradiation resistance attributable to H19 in the context of digestive system cancers have been conducted in vitro. The contribution of H19 to therapy resistance in vivo remains to be further explored. Last but not the least, most gene editing studies on H19 are still preclinical; relevant long-term adverse reactions need to be further explored prior to its clinical application. Future studies are required for in-depth characterization of the involvement of H19 in mediating therapy resistance of digestive system cancer and its underlying mechanisms.

## Data Availability

Not applicable.

## Abbreviations

lncRNAs: Long non-coding RNAs
micro RNA: MiRNA
MDR: Multidrug resistance
EMT: Epithelial–mesenchymal transition
HCC: Hepatocellular carcinoma
GEM: Gemcitabine
5-FU: 5-Fluorouracil
DOX: Doxorubicin
MDR1: Multidrug resistance-associated protein 1
P-gp: P-glycoprotein
IC50: 50% Inhibition concentration
ZEB1: Zinc finger E-box binding homeobox 1
EC: E-cadherin
GST-II: Glutathione *S*-transferase-II
PSEN1: Presenilin 1
CRC: Colorectal cancer
CAFs: Carcinoma-associated fibroblasts
MRP1: Multidrug resistance-associated protein 1
BCRP: Breast cancer resistance protein
SIRT1: Silent information regulator 1
PDAC: Pancreatic ductal adenocarcinoma
ABC: ATP-binding cassette
ESCA: Esophageal carcinoma
GC: Gastric cancer
FADD: Fas-associated death domain
CCA: Cholangiocarcinoma
Twist1: Twist-related protein 1
